# Merging the Worlds of Pets and Pills: Development and Evaluation of an Interprofessional Activity Linking Pharmacy and Veterinary Medicine

**DOI:** 10.1007/s40670-024-02060-6

**Published:** 2024-05-09

**Authors:** Morgan Hoeft, Jared Van Hooser, Nichole Rupnow, Christina Clarkson

**Affiliations:** 1 Department of Pharmaceutical Care and Health Systems, University of Minnesota College of Pharmacy, 3-150C Weaver Densford Hall, 308 Harvard St SE, Minneapolis, MN 55455 USA; 2 Department of Pharmacy Practice and Pharmaceutical Sciences, University of Minnesota College of Pharmacy, Duluth, 217 Life Science, 1110 Kirby Drive, Duluth, MN 55812 USA; 3 Pharmaceutical Care and Health Systems, University of Minnesota College of Pharmacy, 3-150D Weaver Densford Hall, 308 Harvard St SE, Minneapolis, MN 55455 USA; 4 Department of Veterinary and Biological Sciences, University of Minnesota College of Veterinary Medicine, 235D AS/VM Building, 1988 Fitch Avenue, Saint Paul, MN 55108 USA

**Keywords:** Veterinary medicine, Pharmacy, Interprofessional activity, Asynchronous

## Abstract

Interprofessional education (IPE) is noticeably lacking between pharmacy and veterinary medicine students despite the two health professions overlapping in practice. To address this, an asynchronous IPE activity was developed together with practicing pharmacists and veterinarians. Students worked in groups across two campuses to discuss clinical cases, specifically requiring input from both professions. Students reported they learned more about the two professions’ interconnection, felt prepared to interact with the other professionals in the future, and found value in learning from each other. Overall, the results of this study outline a successful pilot IPE activity between veterinary medicine and pharmacy students.

## Background

The World Health Organization states that interprofessional education (IPE) “occurs when students from two or more professions learn about, from and with each other to enable effective collaboration and improve health outcomes” and that IPE “is a necessary step in preparing a ‘collaborative practice-ready’ health workforce that is better prepared to respond to local health needs” [1].

One of the most common interprofessional interactions between veterinarians and other healthcare professionals is with pharmacists [2]. Yet, pharmacists often lack knowledge about the processing and dispensing of veterinary prescriptions [3]. Veterinary pharmacy is not required in pharmacy school curricula by the pharmacy professional program accrediting body [4], and a basic introduction to veterinary medicine is often left to pharmacy elective courses [5]. Practicing community-based pharmacists and veterinarians have demonstrated a strong interest in collaboration; however, they have expressed different viewpoints regarding the pharmacist’s roles in caring for veterinary patients [6].

Both the National Association of Boards of Pharmacy and the American Veterinary Medical Association have recognized the need for improved education about the opposite professions and have called for the development of professional program curricula and the availability of continuing education [7, 8]. There have been calls for further development and assessment of IPE activities between these professional programs [5, 9, 10]. However, IPE activities between veterinary medicine and pharmacy students are lacking [9]. Veterinary pharmacy is an emerging field within the field of pharmacy and requires appropriate interprofessional collaboration [11, 12]. Literature suggests that teaching healthcare students in silos, separated from other professions, may amplify differences between professions and heighten barriers to interprofessional collaborative practice [13, 14]. Ergo, developing and evaluating an IPE activity between these two professions can help prepare students to gain valuable practice in interacting with each other prior to licensure.

At the University of Minnesota, even with an academic health center, there are significant challenges in bringing students across disciplines together to create and implement valuable interprofessional learning activities. The programs’ full schedules with coursework and students’ co-curricular activities limit the availability to bring students and facilitators together face-to-face. Even before the COVID pandemic, there was a need for a creative design to bring the two professions together outside of the classroom. The objective of this pilot IPE activity was to design, implement, and evaluate a remote interprofessional patient care activity with realistic case scenarios involving pharmacy and veterinary medicine students at the UMN to help connect these two professions for further IPE involvement.

### Activity Description

#### Study Design

Four case scenarios were developed by practicing pharmacists and veterinarians to highlight the interplay between pharmacy and veterinary medicine and allow students to improve teamwork and interprofessional collaboration skills. These case scenarios were multifaceted, designed to enrich students’ knowledge of the other profession and to demonstrate how collaboration can promote quality patient care. The case scenarios, vetted by current practitioners, included topics relevant to both professions, such as the opioid epidemic, antibiotic resistance, drug dosing, and appropriate drug information resources (Table 1). The objectives of this activity are highlighted in Appendix 1.

**Table 1:** Description of case scenarios, relevant topics, and case questions

**Scenario 1** Summarized **Case Scenario**: A farmer with dairy cows notices a few of his calves present with coughing and he is low on time and resources. He decides to purchase an over-the-counter antibiotic to treat the calves and roughly estimates a dose instead of visiting a veterinarian to help treat the calves. **Relevant Topics**: Antibiotic stewardship, the over-the-counter status of veterinary products, appropriate dosing of medications in animals Questions: 1. Describe antibiotic misuse and overuse from your profession’s perspective. Why is it a concern? What are the downstream effects of inappropriate use of antibiotics? 2. How do you feel about the availability of antibiotics “over the counter” or available for use without a prescription? What could have been done differently in this scenario? 3. Per state law, prescriptions (a little different than the above scenario) that are used for production animals (i.e. dairy and beef cattle) have a labeling requirement for how long an antibiotic remains in the system of the animal (withdrawal time). Why is this important? How might this be determined? Would you expect it to be the samebetween different cattle, for example?
**Scenario 2** Summarized **Case Scenario: **An owner and his dog with no apparent illness to a veterinary clinic and the owner insists the dog is in pain despite the dog appearing to have no signs of discomfort or pain on physical examination. The owner demands something to treat the pain. **Relevant Topics**: Opioid epidemic, dispensing of medications in a veterinary office, controlled substance laws Questions: 1. What is your understanding of the opioid crisis? Be prepared to bring and share one statistic you find alarming about the opioid crisis. Why did you choose this statistic? 2. Review the short article from the Drug Enforcement Administration about drug diversion. What tactics do you see in this scenario? 3. What other methods of drug diversion could happen in small animal clinic? How about a pharmacy? What safeguards are employed to help prevent or catch drug diversion? 4. What are the regulations for storing controlled substances (e.g., anything with a DEA classification of schedule I-V, like tramadol) in a vet clinic? In a pharmacy? After discussing, highlight the differences and similarities. 5. In your respective curriculums, what do you learn about opioids?
**Scenario 3** Summarized **Case Scenario**: A pharmacist is verifying and filling a new prescription that turns out to be for a dog. The pharmacist is required to dose the medication and compound a prescription dosage form as the dosing for the pet is outside the currently available formulations. **Relevant Topics**: Compounding pharmaceutical dosage forms, legal requirements surrounding compounding, appropriate dosing for animals, educating patients on tips for administering medications to pets Questions: 1. Per state law, what needs to be on a prescription written for an animal to make it a legal prescription? What does Minnesota law say about compounding for animals? What are the compounding requirements in general? 2. What dose of enalapril would be appropriate for a dog? Where would you look to find this information? Are there any important considerations to think about for dosing this medication? 3. Pharmacy students - please explain the IESC framework when considering the appropriateness of medications. How would you consider if this was appropriate? Who would you contact if you were not sure? Have a discussion about how a phone call like this might go, and how to promote interprofessional communication while doing it. 4. What type of counseling would you provide to the owner? What would be important from his perspective? 5. Why does this medication require compounding? Briefly discuss how the compound would be made. Why might a pharmacy be the place better suited for this compound to be made? Can it legally be made in a veterinary clinic?
**Scenario 4** Summarized **Case Scenario**: A pharmacist is asked to assist with selecting an over-the-counter medication to help treat a sick cat despite the owner not having the pet assessed by a veterinarian. **Relevant Topics**: Appropriate care, diagnosis, and exclusion criteria for over-the-counter products for animals, appropriate resources for answering animal-based drug information questions Questions: 1. How would you determine the dose of ibuprofen for this cat? 2. How is ibuprofen metabolized in a human? How is it metabolized in a cat? Is the process the same, or are there differences? 3. Much like in human medicine, there are websites that are available to purchase medications for pets online, sometimes without a prescription. Is this always safe? Are there certain over-the-counter medications that may be appropriate for a human, but completely inappropriate for a pet? Think of conditions when you would not want your patients purchasing medications online without professional oversight. 4. What references can be used to look up dosing for animals? 5. What would be your response to the man in the scenario? Thinking about your profession, consider how this might be resolved interprofessionally. While answering this question, also consider your scope of practice. Is it appropriate to answer questions or offer advice to patients (humans or pets) that is outside your scope of practice?

Third-year veterinary medicine students at the UMN Twin Cities and second-year pharmacy students at the UMN Twin Cities and Duluth campuses were divided into interprofessional and intercampus groups of 6–7 students, with each group having students from veterinary medicine and pharmacy across two campuses. Students were given 6 weeks to schedule their own meetings and complete the activity outside of regularly scheduled class time.

Students started with individual pre-work, which consisted of learning about the curriculum and scope of practice of the profession and reviewing the cases, before meeting virtually as a group (Appendix 2). Then, aligning with a social constructivist approach, the interprofessional student teams met once via a web-based video chat (e.g., Google Hangouts, FaceTime) to discuss the pre-work and work through the case scenarios from their profession’s perspective. Teams completed the case questions as a group and submitted their responses to the learning management system. Individually, students were required to complete an online post-activity survey to receive credit. Approximately 2 h was needed to complete all parts of the activity. Submissions were graded by a teaching assistant on completeness, appropriateness, and on-time submission.

### Data Collection

After completing the activity, pharmacy and veterinary medicine students received a link by email to complete an online survey via Qualtrics (Provo, UT). The survey was composed of a 5-point Likert scale (1 = strongly disagree, 5 = strongly agree) to evaluate students’ perceptions of what they learned about the interconnection between the professions, the value of learning about the other healthcare professionals, and their preparedness to interact with other healthcare professionals. In addition, students responded to several open-ended questions and a yes/no question assessing the enjoyment of the activity.

### Data Analysis

Descriptive statistics, including frequency and percentages, were used to analyze the survey results. Likert items were examined by Mann-Whitney *U* tests and a chi-square test was completed for the polar question. *P*-values < 0.05 were considered statistically significant in all tests, and JMP Pro 14 (SAS Institute Inc., Cary, NC) was used for analysis. Open-ended questions were reviewed for common themes.

## Results

In the spring 2019 semester, 97 veterinary medicine students and 166 pharmacy students were assigned to teams to complete the IPE activity. Ninety-seven veterinary medicine and 156 pharmacy students completed the survey (100% and 95.7% response rates, respectively). Table 2 displays student agreement to prompts on a Likert scale by profession. In response to the prompt “I learned more about the interconnection between pharmacy practice and veterinary medicine practice,” 74% of pharmacy students and 75% of veterinary medicine students somewhat agreed and agreed. To the prompt “I feel prepared to interact with other healthcare professionals in the future,” 81% of pharmacy students and 83% of veterinary medicine students somewhat agreed and agreed. In response to the prompt “I found value in learning from the pharmacy/veterinary medicine students,” 84% of pharmacy students and 73% of veterinary medicine students somewhat agreed and agreed. There was no statistical difference between veterinary medicine and pharmacy student responses regarding these three prompts (*p* = 0.677, *p* = 0.619, and *p* = 0.052, respectively). Over half, 56% and 52%, of pharmacy and veterinary medicine students, respectively, reported the activity to be fun. There was no difference in reported fun between student groups (*p* = 0.666).

**Table 2:** Student responses to Likert items for prompts by profession (*n* = 253)

**Prompt**		**Pharmacy** (*n* = 156)		**Veterinary Medicine** (*n* = 97)	
		*n *=	%	*n *=	%
“I learned more about the interconnection between pharmacy practice and veterinary medicine practice”	Agree	51	32	33	34
	Somewhat Agree	66	42	40	41
	Neutral	28	9	17	18
	Somewhat Disagree	8	4	5	5
	Disagree	6	3	2	2
“I feel prepared to interact with other healthcare/pharmacy/veterinary professionals in the future”	Agree	70	44	45	46
	Somewhat Agree	59	37	36	37
	Neutral	20	13	12	12
	Somewhat Disagree	8	8	2	2
	Disagree	2	1	2	2
“I found value in learning from the pharmacy/veterinary medicine students”	Agree	78	49	39	40
	Somewhat Agree	56	35	32	33
	Neutral	14	9	14	14
	Somewhat Disagree	7	4	5	5
	Disagree	4	3	7	7
“I found this activity to be fun”	Yes	88	56	50	52
	No	69	44	47	48

Positive themes that emerged from the open-ended questions were being able to learn about the other professions from students entering that profession and increasing understanding of how the other profession approaches patient issues and solves problems related to patient care. Negative themes included balancing different class schedules amongst all group members and difficulty via web-based video platforms.

## Discussion

Overall, the results from this interprofessional pilot activity were positive. This pilot activity successfully allowed students from two professions, across two different campuses, to connect and learn more about each other’s profession. Literature has suggested that while students recognize the value of IPE, there is often discontent with the implementation of IPE activities [15]. In this pilot, over half of the students found the activity to be fun. Interestingly, over three-fourths of the students agreed or somewhat agreed that they found the experience valuable, implying that the activity, although not fun for all, was found to be valuable to a large portion of students. Additionally, it was reassuring that there were no statistical differences between the two groups in all questions.

Advantages of this novel interprofessional activity include the ease of implementation, limited administrative burden/oversight required, and no additional costs to programs. Development of the cases and vetting of the case scenarios required the most coordination and time for development; however, once created, minimal updates are required annually. Further, integration of this activity into the course required no additional TA support hours or instructors for grading the assignments. Thus, it can likely be implemented and sustainable at other institutions with ease. Despite calls for further development and assessment of IPE activities and the backing of professional organizations, there is a scarcity of IPE literature between these specific two professions working together in practice and a paucity of IPE literature in pre-licensure pharmacy and veterinary students working together. Therefore, there is a necessity to disseminate successful projects like this to encourage and inspire others to start to address this unmet need. This innovative, asynchronous, IPE activity could feasibly be adapted by other institutions looking to connect these two professions.

The main limitation is that the data was limited to a single cohort of students. However, the activity has been successfully completed during four separate semesters, indicating its sustainability. There are opportunities to strengthen this activity in the future. From a student perspective, the primary suggestion was to improve the logistics of the assignment, specifically regarding the need for a university-sponsored video conferencing platform to assist students in connecting with their peers to complete the assignment. Fortunately, Zoom (San Jose, CA) was adopted by the UMN and is available for students to use to bridge this connection gap. Students further suggested coordinating across pharmacy and veterinary medicine to find a time to mutually be excused from class to help facilitate finding a time for groups to meet. This could be considered to assist students in finding time to complete the group work required for this assignment. Additionally, it may be beneficial to continue building on the IPE experience and include a debrief class session between both pharmacy and veterinary medicine students to allow for deeper discussion and team-building between pharmacy and veterinary medicine students. As a result, an activity of this structure could be implemented to connect pharmacy students to other health professionals that historically have lacked coordinated interprofessional education opportunities, such as dental hygiene. Lastly, future renditions of this activity may include higher-order IPE assessments and including an integrated class session with both professions working together on common cases in real-time.

As pharmacists and veterinarians may likely experience opportunities for collaboration in practice, designing an activity with a clinical application that draws on the availability of two professional programs is novel and desirable. This pilot aimed to design, implement, and evaluate an interprofessional patient care activity involving pharmacy and veterinary medicine students. Based on the results of the survey and student responses, this online, asynchronous pilot is an effective IPE activity, bridging two professions across two campuses. Findings were overall positive from both cohorts of students, with most feeling better prepared to interact with other healthcare professionals in the future.

## Data Availability

The authors confirm that the data supporting the findings of this study are available within the article.

## Appendix 1. Objectives of the Activity

1. Establish interprofessional skills by creating relationships that can be useful in practice.
2. Acknowledge differences in practice models, patient populations, and standards of care between pharmacy and veterinary practices.
3. Understand where influence from other healthcare professionals can be appreciated and can promote more patient-centered care, regardless of the species of the patient.

## Appendix 2. Individual Pre-work Questions

### Pre-work Questions for Pharmacy Students

1. How many years does a veterinarian go to school?
2. When graduating from veterinarian school, what level of degree is obtained?
3. What is the scope of practice for veterinarians? List potential career options.
4. Is post-doctoral training available and/or required? If so, what does it look like?
5. Can veterinarians prescribe medication? Can they dispense?
6. How many semesters of pharmacology and/or medicinal chemistry are required in their curriculum?

### Pre-work Questions for Veterinary Medicine Students

1. How many years does a pharmacist go to school?
2. When graduating from pharmacy school, what level of degree is obtained?
3. What is the scope of practice for pharmacists? List potential career options.
4. Is post-doctoral training available and/or required? If so, what does it look like?
5. Can pharmacists prescribe medication?
6. How many semesters of pharmacology and/or medicinal chemistry are required in their curriculum?
